# Retraction: MicroRNA-101-3p inhibits fibroblast-like synoviocyte proliferation and inflammation in rheumatoid arthritis by targeting PTGS2

**DOI:** 10.1042/BSR-20191136_RET

**Published:** 2020-08-04

**Keywords:** Rheumatoid arthritis, microRNA-101-3p, Prostaglandin-endoperoxide synthase 2, Fibroblast-like synoviocytes, Synovial injury

This article is being retracted from *Bioscience Reports* at the request of the Editor-in-Chief and the Editorial Board following receipt of a notification from a reader, alerting the Editorial Board to manipulation in Figure 8C.

The authors have been contacted in regard to the retraction, and have not responded to the Journal. Given the extent of the issues raised, Editorial Board stand by the decision to retract the article.

